# Radiological and Embryological Relevance of Persistent Sciatic Arteries: A Rare Presentation

**DOI:** 10.7759/cureus.18660

**Published:** 2021-10-11

**Authors:** Varna Taranikanti, Jnana Aditya Challa, Alok Kumar Mittal

**Affiliations:** 1 Foundational Medical Studies, Oakland University William Beaumont School of Medicine, Rochester, USA; 2 Interventional Radiology, Sultan Qaboos University Hospital, Muscat, OMN

**Keywords:** superficial femoral artery, persistent sciatic artery, embolization, anomaly, aneurysm

## Abstract

Persistent sciatic artery (PSA) is an extremely rare condition that is present in around 0.05% of the population and is commonly associated with many complications. The management is conservative or through surgical intervention and depends on the type of complication. The case presented is of a 40-year-old man who complained of persistent pain in the buttock region. On radiology, bilateral PSAs were observed exiting through the infra-pyriformis compartment of the greater sciatic foramen accompanying the sciatic nerve. The femoral artery and the external iliac artery are small in caliber. In this case report, we discuss the underlying embryology that might have led to the persistence of this vessel with illustrations and the abnormal radiological pattern of this anomaly. Increased awareness of PSA can improve patient care and prevent potentially hazardous complications during hip and renal transplant surgery.

## Introduction

Persistent sciatic artery (PSA) is an extremely rare vascular anomaly resulting from a lack of regression of an embryonal artery to the lower extremity. It is present in around 0.05% of the population, and only around 49 cases have been published in the world literature since 1832 [1]. It originates from the internal iliac artery, courses close to the sciatic nerve, and provides the main supply to the popliteal artery. It is commonly seen on the right side; however, 20% of patients with this vascular anomaly present bilaterally with no sexual preponderance [2]. Patients commonly present with buttock pain in their adult years and hence it is found incidentally during routine evaluation of patient’s symptoms. PSA is particularly prone to undergo aneurysm formation or atherosclerosis. Some of the other congenital abnormalities associated with the persistence of this vessel are abnormal right subclavian artery, arteriovenous fistulas, Mullerian agenesis, and atypical patterning of varicose veins [3]. A literature search resulted in one systematic review, which revealed that 48% of cases presented with aneurysm of the PSA [4].

This anomaly should be kept in mind in the clinical assessment of a pulsatile gluteal mass as it presents potentially hazardous complications during hip and renal transplant surgery [1]. In this case report, we discuss the underlying embryology that relates to the persistence of the embryonic axial artery with illustrations and the abnormal radiological pattern of this anomaly.

## Case presentation

**Figure 1:**
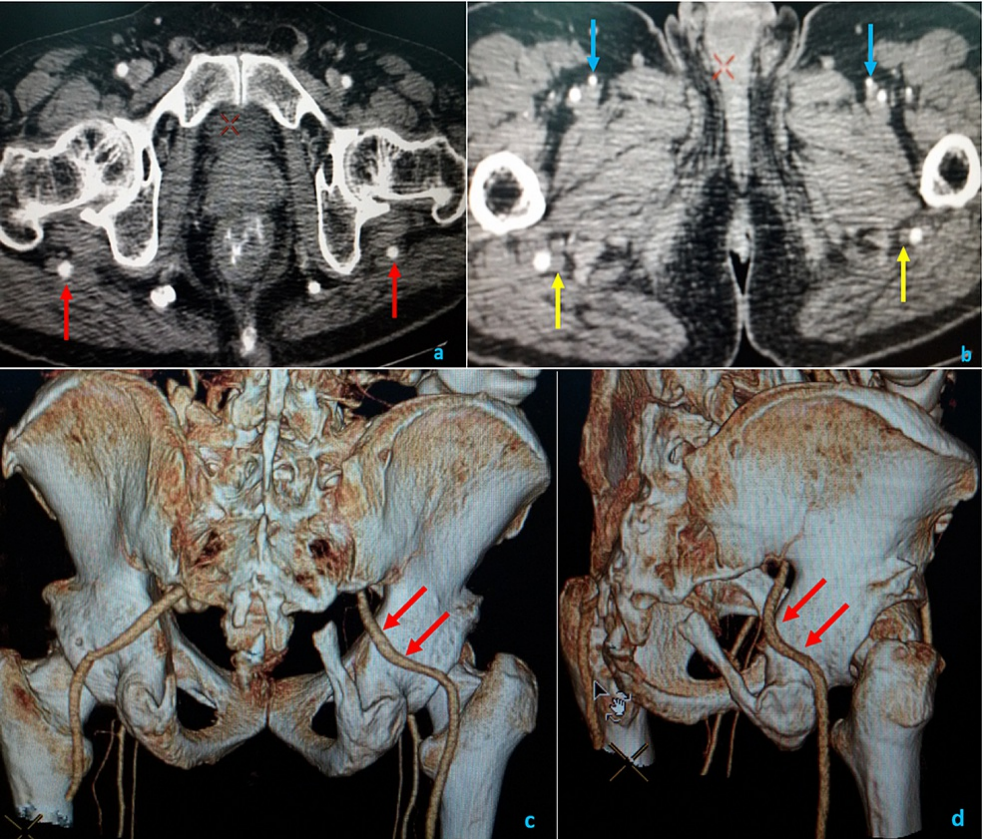
A persistent sciatic artery in a 40-year-old male patient CT angiogram and volume-rendered images showing ischial tuberosity (a), upper thigh (b), posterior projection (c), and oblique projections (d). Also seen are axial limb arteries (red arrows). Arteries are accompanying the sciatic nerves (yellow arrows), bilateral superficial femoral arteries (blue arrows), and persistent sciatic artery (red arrows).

A 40-year-old man underwent a CT angiogram (CTA) investigation due to a six-month history of pain in the buttock region, which showed bilateral arteries exiting through the infra-pyriformis compartment of the greater sciatic foramen accompanying the sciatic nerve. The axial CTA images taken at levels of ischial tuberosity (Figure 1a) and upper thigh (Figure 1b) showed these arteries (Figure 1 ) as a continuation of the internal iliac arteries, exiting bilaterally into the posterior thigh through the infra-pyriformis compartment of the greater sciatic foramen. The arteries exit about 1cm from the greater sciatic foramen bilaterally. They were about 5mm in caliber and continued as the popliteal arteries close to the sciatic nerve (Figure 1 ). Volume-rendered CT imaging showed posterior and oblique projections (Figures 1c, 1d) confirming the continuation of internal iliac arteries as PSAs, exiting through greater sciatic foramen bilaterally (Figure 1). The CT also showed the relatively smaller-sized bilateral superficial femoral arteries ([SFAs] Figure 1 ).

## Discussion

**Figure 2:**
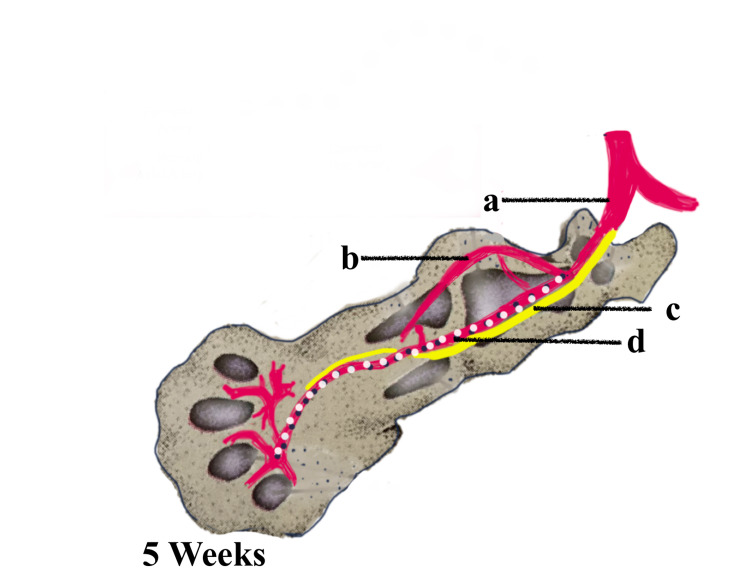
Developing limb with the axial artery continuing as the sciatic artery (a) Common Iliac artery, (b) Internal Iliac artery. (c) Sciatic nerve. (d) Sciatic artery. Image created by Dr. Taranikanti using Sketchbook software (https://www.sketchbook.com)

**Figure 3:**
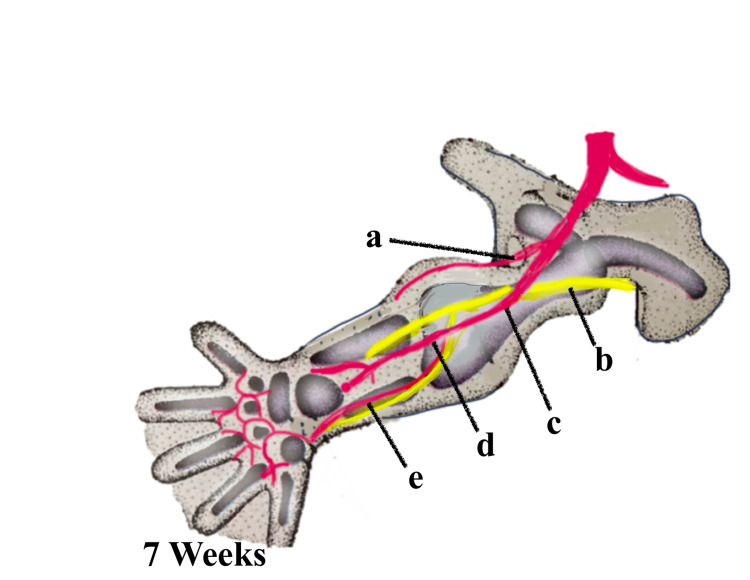
Persistence of the sciatic artery into the entire limb with the superficial femoral artery of a smaller caliber (from the external iliac artery) (a) Internal iliac artery. (b) Sciatic nerve. (c) External iliac artery. (d) Poplitial artery. (e) Posterior tibial artery. Image created by Dr. Taranikanti using Sketchbook software (https://www.sketchbook.com)

**Figure 4:**
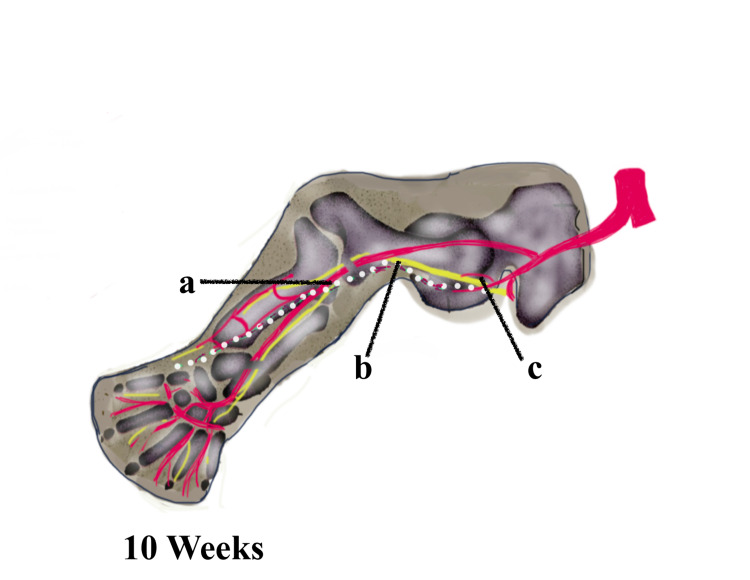
Regression of the sciatic artery with the persistence of the superficial femoral artery (normal vascular pattern of the limb) (a) Obliterated sciatic artery. (b) Sciatic nerve. (c) Arteria nervi ischiadica. Image created by Dr. Taranikanti using Sketchbook software (https://www.sketchbook.com)

PSA, especially the bilateral variant, is a very rare congenital vascular anomaly due to the abnormal developmental process of the axial limb vasculature. Knowledge of the embryological patterning of the lower limb vessels, which involves vasculogenesis, angiogenesis, sprouting, and resorption, is essential for understanding the persistence of the sciatic artery. The major arteries of the body wall and limbs are derived from the intersegmental arteries. Around 30 or more pairs arise serially at each segmental level and run between the somites [5]. In the limbs, the intersegmental arterial pattern matures in a proximal-distal direction in concert with the skeletal and muscular elements of the developing limb. In the abdominal region, the fifth lumbar intersegmental artery continues as the embryonic common iliac artery. This branches into the embryonic internal and external iliac arteries. The first major artery to penetrate the lower limb is the branch of the embryonic internal iliac artery called the central axial artery or sciatic artery, which terminates at the distal end of the limb as a capillary network, as seen in Figure 2. Shortly thereafter, the embryonic femoral artery buds by angiogenesis from the external iliac artery and joins the proximal portion of the sciatic artery and eventually lies parallel to the axial sciatic artery distally and terminates in the foot [6]. During this process, the axial artery undergoes major alterations leading to a degeneration of the distal portion of the artery, as seen in Figure 3. Sometimes, due to failure of degeneration, this axial sciatic artery may become the dominant artery, which may become the sole supply to the lower limb, as seen in Figure 4. In such a case, the SFA and the profunda femoris artery may present as very small branches. The axial sciatic artery may continue as the popliteal artery where it is called a complete sciatic artery occurring in more than 80% of anomalies or it may be incomplete where the main artery remains connected through collateral vessels [7].

A literature search revealed that PSAs are classified into five types according to the Pillet-Gauffre classification. Type 1 represents complete PSA with normal SFA, type 2 represents complete PSA with incompletely developed SFA, and type 3 represents incomplete PSA with the persistence of proximal SFA. Type 4 represents complete development of SFA but the axial artery (represents the sciatic artery) is persistent at the lower part. Type 5 is a mix of the above [8]. The axial sciatic artery continues as the popliteal artery where it is known as the complete sciatic artery as seen in Pillet-Gauffre classification system 1 and 2. However, in our case in addition to this, the femoral artery is smaller in caliber, making it fall into the type 2 category of this classification system. Because the SFA is hypoplastic in this case, it contributes only collaterals to the knees.

As seen in the radiological images, these arteries are located superficially and need to be further evaluated as they tend to result in pathologic vascular complications such as aneurysm formation. Symptoms such as lower limb pain, claudication, cold lower extremities, black toes, neurological deficits (sensory and motor), and pain along sciatic nerve distribution are some of the clinical presenting signs and symptoms of this anomaly. A minority of patients also present with a palpable popliteal pulse in the absence of femoral pulse (Cowie’s sign) [3]. This anomalous vessel that runs superficially in the buttock region may cause repeated trauma during flexion and extension of the hip joint during sitting and standing positions and can result in atheroma and aneurysm formation.

The management of these types of cases is variable depending on the pathology as well as the classification type. While types 1 and 2 are typically treated medically, arteries in the other types are prone to embolism and aneurysm formation and are treated by interventional radiologists with coil embolization [8].

## Conclusions

PSA is an extremely rare condition and is commonly associated with many complications. In this case, bilateral PSAs were observed exiting through the infra-pyriformis compartment of the greater sciatic foramen accompanying the sciatic nerve. The management is dependent on the type of complication. As the patient in our case presented with pain in the gluteal region, and the CT failed to reveal any aneurysm or embolism, he was managed conservatively with pain control medications.
